# Considering the use of GLP-1 receptor agonists in women with obesity prior to pregnancy: a narrative review

**DOI:** 10.1007/s00404-024-07849-9

**Published:** 2025-01-07

**Authors:** Sarah A. L. Price, Alison Nankervis

**Affiliations:** 1 Department of Medicine, Royal Melbourne Hospital, University of Melbourne, Grattan St, Parkville, VIC 3050 Australia; 2 Department of Obstetric Medicine, Royal Women’s Hospital, Flemington Rd, Parkville, VIC Australia; 3 Department of Diabetes and Endocrinology, Royal Melbourne Hospital, Grattan St, Parkville, VIC Australia

**Keywords:** Pregnancy, Obesity, GLP-1 RA, Weight loss, Pregnancy outcomes

## Abstract

**Purpose:**

Metabolic disease, including obesity and type 2 diabetes, are amongst the most significant health issues facing women of reproductive age. To date, no antenatal weight management tools have reduced the risk of adverse health outcomes for women with obesity and their offspring, resulting in a shift in focus to the pre-conception period. Although not yet recognised in most international weight management guidelines, glucagon-like peptide-1 receptor agonists (GLP-1 RAs) are being increasingly used for weight management prior to conception.

**Methods:**

A literature search of PubMed, Medline, and Embase databases identified relevant articles describing the use of GLP-1 RAs prior to and during pregnancy. Papers were selected based on relevance and originality, with clinical trials, large observational studies and meta-analyses being preferentially included.

**Results:**

This narrative review summarises the mechanism of action of GLP-1 RAs and the clinical effects observed in non-pregnant adults. It synthesises the available data from human and animal studies regarding the safety and efficacy of GLP-1 RAs prior to pregnancy, and the consequences of inadvertent drug exposure in early pregnancy. In considering the need to balance the risks of metabolic disease with the risks posed by inadvertent drug exposure, it highlights the areas where further research is needed to guide clinical decision-making.

**Conclusion:**

GLP-1 RAs may have a role in facilitating weight loss and improving the metabolic health of women prior to pregnancy. However, there is currently insufficient evidence to demonstrate that the use of this class of drugs prior to pregnancy improves pregnancy outcomes.

## Introduction

Obesity is the most common health issue impacting women of reproductive age [1–3]. It significantly increases the risk of adverse pregnancy outcomes such as congenital anomalies, preeclampsia, abnormal fetal growth, preterm delivery and pregnancy loss [4–6] Preterm delivery, especially if it is very preterm or extremely preterm, and major congenital anomalies can result in children living with long term disabilities and chronic disease [7–9]. Women who have metabolic disease in pregnancy have an increased long-term risk of major cardiovascular events, in part because pregnancy unveils underlying endothelial dysfunction [10, 11].

Some countries and major scientific organisations are moving from defining obesity as ‘lifestyle choice’ to a ‘disease’ to reflect the biological basis of this condition [12, 13]. Although not universally accepted, there is broad international agreement that people with obesity should be supported through good clinical care and evidence-based guidelines, and that health policy should be aimed at reducing stigma and promoting health equity [14]. This is particularly true in women planning pregnancy where the metabolic status of the mother is thought to impact the long-term health of the offspring [15].

Thus far, antenatal weight management trials, including the seminal LIMIT study, have failed to show an improvement in pregnancy outcomes for women with obesity [16, 17]. This resulted in a shift in focus to interventions in the pre-conception period, which coincided with the introduction of glucagon-like peptide-1 receptor agonists (GLP-1 RAs) into routine clinical practice. This class of drugs improves glycaemic control without hypoglycaemia and achieves substantial weight loss by addressing appetite, likely representing the most significant advance in the care of people living with metabolic disease this century [18, 19].

The use of GLP-1 RAs by women with obesity who are planning pregnancy is controversial [20–22]. Some clinicians argue that these drugs pose an unquantifiable risk to the mother and unborn child due to a lack of trial data in the population of interest [23]. However, uncontrolled metabolic disease during pregnancy accelerates cardiovascular disease complications in the mother and increases the risk of congenital defects, death, and disability in the offspring [7, 24, 25]. To avoid these devastating outcomes, there is an urgent need to determine if GLP-1 RAs have a role in women with metabolic disease planning pregnancy.

## Guidelines for pre-pregnancy weight management in women with obesity

The guidelines from five international maternity societies (American College of Obstetrics and Gynaecology (ACOG); Society of Obstetrics and Gynaecology Canada (SOGC); International Federation of Obstetrics and Gynaecology (FIGO); Royal Australian and New Zealand College of Obstetrics and Gynaecology (RANZCOG); Royal College for Obstetrics and Gynaecology (RCOG)), all support pre-conception weight loss in women with obesity, defined at a body mass index (BMI) > 30 kg/m^2^). However, no guidelines specify the optimal amount of weight loss [26]. All guidelines recommend lifestyle management including diet and exercise, but only the SOGC guideline currently considers the use of pharmacological therapies to induce weight loss prior to conception [27].

For women requiring assisted fertility, some organisations restrict the availability of fertility treatments to women above certain BMI thresholds [28, 29]. This is controversial given that the data favouring BMI thresholds is sparse, and amount of weight loss achieved may be more important than the absolute body weight. Other factors such as maternal age [30] and co-morbidities must also be considered. Furthermore, BMI thresholds which often require women to lose massive amounts of weight to qualify for fertility treatments are major factors driving to use of GLP-1 RAs prior to pregnancy [31, 32].

Globally, increased use of GLP-1 RA’s is occurring in women planning pregnancy despite Federal Drug Administration (FDA) advice that it is unknown if this class of drug ‘*will harm your unborn baby’* [22]. Similarly, the Therapeutic Good Administration (TGA) has classed these medications as category D (*drugs which have caused, are suspected to have caused or may be expected to cause, an increased incidence of human fetal malformations or irreversible damage*) [33]. Currently, women are advised to cease these drugs two months prior to conception based on pharmacokinetic studies [22, 34]. There are no studies that explore the consumer perspective to understand the rationale of women using these drugs in the pre-conception period.

### Endogenous incretin hormones

Endogenous GLP-1 is produced from proglucagon expressed in the gut, pancreas and brain, which is subsequently cleaved in the intestinal L cells to form glucagon and GLP-1 [35–37]. The GLP-1 receptor is expressed in the gut, pancreas, brainstem, hypothalamus and vagal afferent nerves [35]. Ingestion of nutrients, especially fat and carbohydrates, stimulates GLP-1 secretion [37]. Activation of the GLP-1 receptor in the gut delays gastric emptying and in the brain increases satiety [35]. Both result in reduced food intake. In the pancreas, GLP-1 receptor activation increases glucose-dependent insulin release and decreases glucagon secretion, facilitating glucose homeostasis [18, 38, 39].

Endogenous GIP (Glucose-dependent Insulinotropic Polypeptide) is synthesised in the K cells of the duodenum [40]. The GIP receptor is expressed on the alpha and beta cells of the pancreas, gut, adipose tissue, brainstem, hypothalamus and vagal afferent nerves. Like GLP-1, GIP receptor activation induces satiety and stimulates insulin secretion in a nutrient-dependent manner. However, GIP enhances glucagon secretion during hypoglycaemia, while GLP-1 decreases glucagon during hyperglycaemia. GIP also directly stimulates lipogenesis, while GLP-1 indirectly promotes lipolysis [41].

Diet-induced weight loss in non-pregnant human adults’ results in a reduction in fasting GLP-1 and an increase in GIP [42]. This is presumably adaptive, stimulating increased food intake and storage, and driving weight regain [43]. In pregnancy, there is a progressive decrease in GLP-1 across the pregnancy [44] which likely facilitates increased oral intake and allows shunting of fuel to the growing fetus. The impact of pregnancy on GIP is less clear. Given the rapid increase in GLP-1 production in the peripartum period, it is likely that endogenous GLP-1 has activities beyond appetite regulation and glucose homeostasis, but these are still being elucidated [45].

### Mechanism of action of incretin analogues

Incretins are metabolic hormones that stimulate insulin secretion after nutrient intake [46]. Incretin analogues are a family of drugs that mimic the action of endogenous incretin hormones. GLP-1 RAs mimic the action of GLP-1 and act via the GLP-1 receptor, while GIP RAs mimic the action of GIP and act via the GIP receptor [47]. Compared to endogenous incretin hormones which have a very short half-life of a few minutes, exogenous GLP-1 RAs and GIP-RAs have been modified to have a half-life of between 24 h and 7 days depending on the agent [22]. These drugs act in the gut to delay gastric emptying and increase central satiety signalling. They also increase glucose-dependent insulin release and decrease glucagon signalling [47]. GLP-1 RAs may also reduce inflammation both indirectly through weight loss and directly through activation of GLP-1 activation on T cells, which may explain the lower rates of cardiorenal disease in people using these drugs [48].

In non-pregnant adults, large clinical trials of incretin analogues have demonstrated increasing greater efficacy in weight loss and glycaemic control. In 2015, the SCALE study demonstrated that the GLP-1 analogue Liraglutide (3.0 mg weekly) results in 8.0% body weight loss [49], and a mean HbA1c reduction of 1.1% [50] after 56 weeks. In 2021, STEP-1 demonstrated that Semaglutide (2.4 mg weekly) results in 12.4% weight loss over 68 weeks [51], and a mean HbA1c reduction of 1.59% [52]. In 2022 SURMOUNT-1 demonstrated that the GLP-1/GIP analogue Tirzepatide (15 mg weekly) results in 20.9% weight loss over 72 weeks [53] and a mean HbA1c reduction of 1.95% [52]. Although these trials have all included women who inadvertently achieved pregnancy, the trials have specifically excluded women planning pregnancy by requiring women of reproductive age to use two forms of contraception.

GLP-1 RAs are indicated both for weight loss and diabetes control, although prescribing rules vary between countries. While some women may inadvertently become pregnant while using GLP-1 RAs, there is little doubt that others are making a deliberate decision to use these drugs prior to pregnancy [54]. This may be in the context of sub-fertility, such as in women with polycystic ovarian syndrome or anovulation secondary to obesity, or in women who are aiming to lose weight or improve glycaemic control to improve pregnancy outcomes [20, 55]. Importantly, there is no trial evidence to support GLP-1 RAs for this latter indication.

GLP-1 RAs should not be deliberately used in pregnancy, nor would weight loss in pregnancy be desirable [56]. However, given the widespread use of this class of drugs in women planning pregnancy, inadvertent administration in early pregnancy is inevitable. It is presumed that GLP-1RAs do not cross the placenta based on their molecular size. Animal studies regarding this issue are described in the section below. However, use prior to pregnancy or in early pregnancy would be expected to alter the maternal metabolic state and, therefore, both maternal and fetal effects of the drug are possible.

### Human studies of GLP-1 RAs

Cesta et al. published an audit of women inadvertently exposed to GLP-1 receptor agonists in the first trimester of pregnancy and reported no increase in the risk of major congenital malformations (adj RR 0.95 (0.72–1.66), and no increase in the risk of major cardiac malformations (adj RR 0.68 (0.42–1.12)) [54]. Dao et al. explored the reproductive safety of GLP-1 based on the databases of six teratology information services based in Europe and Israel. The 168 women exposed were compared to a reference group with diabetes (*n* = 156) and a reference group with overweight/obesity (*n* = 163). There was no difference in spontaneous abortion, medical termination of pregnancy and stillbirth [57]. Taken together, these studies demonstrate early reassurance about congenital anomalies and fetal death in the context of short-duration exposure in the first trimester of pregnancy.

In a small retrospective study of 188 pregnancies, mean gestational weight gain was greater in women exposed to GLP-1 prior to pregnancy than unexposed women (11.3 ± 5.4 vs 8.6 ± 7.8 kg, 95% CI 0.60–11.1) [58]. This suggests that pre-pregnancy GLP-1 RA use is associated with an additional ~ 3 kg gestational weight gain. However, previously reported studies using both lifestyle modification and a Very Low Energy Diet (VLED) report gestational weight gain of ~ 3 kg after pre-pregnancy weight loss [59]. Therefore, it is likely that weight loss, and not the drug per se, is driving the additional gestational weight gain. This is further supported by data from Purcell et al. which demonstrates that the rate of weight loss does not impact the rate of weight regain [60].

There are no human studies of GLP-1 RA in lactation. However, transfer to human milk is likely to be low given that GLP-1 RAs are large peptide molecules [61]. Furthermore, GLP-1 ingested by human infants is likely to be largely digested in the gastrointestinal tract of the infant [23].

### Animal studies of GLP-1 RAs

Muller et al. published a systematic review of animal studies using GLP-1 RAs in pregnancy [23]. This review included data from the European Medicines Agency (EMA) and Federal Drug Administration (FDA) including product information, the Netherlands Pharmacovigilance Centre and the Teratology Information Service of Switzerland. In rat, mouse and rabbit models, GLP-1 RA administration caused retardation of ossification, skeletal variations, major skeletal anomalies and visceral congenital anomalies in a dose dependent manner [23]. These effects were mostly accompanied by decreased maternal food consumption and reduced maternal weight. Although animal models of malnourished mothers have described delayed ossification and skeletal effects, these malnutrition models do not report visceral anomalies. It is also uncertain if the rates of visceral anomalies are higher than that seen in the population of unexposed animals.

While low molecular weight molecules (< 600 Daltons) cross the placenta freely, larger molecules including GLP-RAs (4000–63,000) are presumed not to cross the placenta based on their molecular size [61]. Extendin-4 is a long-acting GLP-1 analogue that is often used in animal studies [62]. One animal study using Exendin-4 found that when mice were injected with the drug and were sacrificed one day later, there was no drug on the fetal side of the placenta. However, when pregnant mice with systemic inflammation were injected with Exendin-4, there was evidence of the drug on the fetal side of the placenta [63]. This suggests while most of the impact of these drugs in pregnancy may be modulated by on the maternal side of the placenta, direct fetal effects cannot be excluded.

When administered in the second trimester of pregnancy in an animal model, Qiao et al. demonstrated that Semaglutide decreases fetal body weight and placental capillary density [44]. Although placental diameter is not affected, there is reduced vessel branching which results in reduced surface areas for nutrient exchange and reduced blood flow [44]. This placental effect likely explains the increase in fetal growth restriction observed in the animal studies.

It is likely that endogenous GLP-1 has effects on fetal development beyond appetite regulation and fetal growth [48, 64]. GLP-1 receptor appears to be rapidly upregulated in the peripartum period, which correlates with increases in surfactant production and early lung function [45]. The impact of exogenous GLP-1 RA administration on these late pregnancy effects is essentially unknown.

Post-partum, when GLP-1 RAs are administered to animals during lactation, the concentration of exenatide in milk in mice, when compared with the maternal concentrations, were < 2.5% for exenatide, 8.3–33% for Semaglutide and 50% for Liraglutide [23].

### Areas of uncertainty

The evidence that obesity and sub-optimally controlled diabetes adversely affects pregnancy outcomes is over-whelming [65, 66]. However, it is uncertain if the metabolic benefits of GLP-1 RAs used prior to pregnancy are sustained in pregnancy or if these benefits lead to improved pregnancy outcomes. For women with obesity, it is uncertain how much weight loss may be required to improve pregnancy outcomes, and it is uncertain how best to achieve this weight loss while maintaining maternal nutrition.

For women who use GLP-1 RA prior to conception, the ideal time to cease these drugs is unknown. Based on pharmacokinetic studies, women are routinely advised to cease GLP-1 RA’s five half-lives before conception (i.e. the half-life of Semaglutide is 5–7 days, so the drug should be ceased ~ 6 weeks before conception) [22, 34]. However, spontaneous conception is often unplanned [67] and choosing the timing for drug cessation may be difficult [68, 69]. Therefore, understanding the implications of early pregnancy exposure is critical [70].

After pre-conception weight loss, maternal weight, body composition and appetite profile are altered. The data regarding the benefits of pre-pregnancy weight loss are sparse [59]. In addition to determining the optimal amount of weight loss, the impact of weight loss on lean tissue in women planning pregnancy must be determined [71, 72]. Data from bariatric surgery, which achieves weight loss by alteration of appetite hormones including GLP-1, suggest that significant weight loss may benefit the mother but be detrimental for the offspring with an increased risk of small for gestational age offspring and preterm delivery [73, 74]. The long-term implications of iatrogenic prenatal GLP-1 receptor activation are also unknown [75].

### Future direction

There is little data to describe what is driving the huge demand for GLP-1 RAs in women planning pregnancy [54, 76]. It is possible that women with obesity presume pre-pregnancy weight loss will reduce the risk of adverse pregnancy outcomes. Alternatively, the use of GLP-1 RAs in women of reproductive age could simply reflect the rising rates of obesity in this demographic. Future research must focus on two areas. First, we must determine the direct effects of GLP-1 RAs on women planning a pregnancy with an ongoing collection of teratogenicity data in women who inadvertently used GLP-1 RAs during pregnancy. Second, we must determine the indirect effects of GLP-1 RAs with a specific focus on the implications of pre-pregnancy weight loss on pregnancy outcomes including maternal and neonatal body composition [71] and appetite regulation [77].

### Conclusions

There is compelling evidence that metabolic diseases, including obesity, can adversely impact pregnancy outcomes. GLP-1 RA are being widely used in the preconception period, despite a lack of evidence to demonstrate improved pregnancy outcomes. Human studies of inadvertent exposure to GLP-1 RAs in early pregnancy are reassuring, demonstrating no increase in the risk of congenital abnormalities. However, animal studies suggest that drug use could have a deleterious effect on offspring that is primarily mediated by its effect on maternal nutrition. On this basis, women are advised to avoid pregnancy until five half-lives of the drug have elapsed to allow adequate drug wash-out, and to advocate for trials that specifically include women planning pregnancy.

## References

[1] Iacobucci G (2015) Obesity is one of biggest risks to women’s health, says England’s chief medical officer. BMJ 351:h6762 26657560 10.1136/bmj.h6762

[2] Harris E (2023) US obesity prevalence surged over the past decade. JAMA. 330(16):1515 37792409 10.1001/jama.2023.19201

[3] Australian Institute of Health and Welfare (2023) Australia's mothers and babies. http://www.aihw.gov.au/reports/mothers-babies/australias-mothers-babies accessed 18 December 2023: Australian Government

[4] Callaway LK, Prins JB, Chang AM, McIntyre HD (2006) The prevalence and impact of overweight and obesity in an Australian obstetric population. Med J Aust 184(2):56–59 16411868 10.5694/j.1326-5377.2006.tb00115.x

[5] Schummers L, Hutcheon JA, Bodnar LM, Lieberman E, Himes KP (2015) Risk of adverse pregnancy outcomes by prepregnancy body mass index: a population-based study to inform prepregnancy weight loss counseling. Obstet Gynecol 125(1):133–143 25560115 10.1097/AOG.0000000000000591 PMC4285688

[6] Louwen F, Kreis NN, Ritter A, Yuan J (2024) Maternal obesity and placental function: impaired maternal-fetal axis. Arch Gynecol Obstet 309(6):2279–2288 38494514 10.1007/s00404-024-07462-w PMC11147848

[7] Cheney K, Farber R, Barratt AL et al (2018) Population attributable fractions of perinatal outcomes for nulliparous women associated with overweight and obesity, 1990–2014. Med J Aust 208(3):119–125 29438637 10.5694/mja17.00344

[8] Breukhoven PE, Kerkhof GF, Willemsen RH, Hokken-Koelega AC (2012) Fat mass and lipid profile in young adults born preterm. J Clin Endocrinol Metab 97(4):1294–1302 22399507 10.1210/jc.2011-2621

[9] Rasmussen SA, Chu SY, Kim SY, Schmid CH, Lau J (2008) Maternal obesity and risk of neural tube defects: a metaanalysis. Am J Obstet Gynecol 198(6):611–619 18538144 10.1016/j.ajog.2008.04.021

[10] Lee KK, Raja EA, Lee AJ, Bhattacharya S, Norman JE, Reynolds RM (2015) Maternal obesity during pregnancy associates with premature mortality and major cardiovascular events in later life. Hypertension 66(5):938–944 26370890 10.1161/HYPERTENSIONAHA.115.05920

[11] Prodan NC, Schmidt M, Hoopmann M, Abele H, Kagan KO (2024) Obesity in prenatal medicine: a game changer? Arch Gynecol Obstet 309(3):961–974 37861742 10.1007/s00404-023-07251-x PMC10867045

[12] Wilding JPH, Mooney V, Pile R (2019) Should obesity be recognised as a disease? BMJ 366:l4258 31315832 10.1136/bmj.l4258

[13] The Lancet Diabetes Endocrinology (2017) Should we officially recognise obesity as a disease? Lancet Diabetes Endocrinol 5(7):483 28601583 10.1016/S2213-8587(17)30191-2

[14] Luli M, Yeo G, Farrell E et al (2023) The implications of defining obesity as a disease: a report from the Association for the Study of Obesity 2021 annual conference. EClinicalMedicine 58:101962 37090435 10.1016/j.eclinm.2023.101962 PMC10119881

[15] Painter RC, Osmond C, Gluckman P, Hanson M, Phillips DI, Roseboom TJ (2008) Transgenerational effects of prenatal exposure to the Dutch famine on neonatal adiposity and health in later life. BJOG 115(10):1243–1249 18715409 10.1111/j.1471-0528.2008.01822.x

[16] Dodd JM, Turnbull D, McPhee AJ et al (2014) Antenatal lifestyle advice for women who are overweight or obese: LIMIT randomised trial. BMJ 348:g1285 24513442 10.1136/bmj.g1285 PMC3919179

[17] Catalano PM (2018) Reassessing strategies to improve pregnancy outcomes in overweight and obese women. Lancet Diabetes Endocrinol 7(1):2–3 30528219 10.1016/S2213-8587(18)30337-1

[18] Drucker DJ (2024) Efficacy and safety of GLP-1 medicines for type 2 diabetes and obesity. Diabetes Care 47(11):1873–1888 38843460 10.2337/dci24-0003

[19] Giorda CB, Rossi A, Baccetti F et al (2023) Achieving good metabolic control without weight gain with the systematic use of GLP-1-RAs and SGLT-2 inhibitors in type 2 diabetes: a machine-learning projection using data from clinical practice. Clin Ther 45(8):754–761 37451913 10.1016/j.clinthera.2023.06.006

[20] Maslin K, Alkutbe R, Gilbert J, Pinkney J, Shawe J (2024) What is known about the use of weight loss medication in women with overweight/obesity on fertility and reproductive health outcomes? A scoping review. Clin Obes 14:e12690 38951960 10.1111/cob.12690

[21] Minis E, Stanford FC, Mahalingaiah S (2023) Glucagon-like peptide-1 receptor agonists and safety in the preconception period. Curr Opin Endocrinol Diabetes Obes 30(6):273–279 37678163 10.1097/MED.0000000000000835 PMC10615799

[22] Drummond RF, Seif KE, Reece EA (2024) Glucagon-like peptide-1 receptor agonist use in pregnancy: a review. Am J Obstet Gynecol. 10.1016/j.ajog.2024.08.024 39181497 10.1016/j.ajog.2024.08.024

[23] Muller DRP, Stenvers DJ, Malekzadeh A, Holleman F, Painter RC, Siegelaar SE (2023) Effects of GLP-1 agonists and SGLT2 inhibitors during pregnancy and lactation on offspring outcomes: a systematic review of the evidence. Front Endocrinol (Lausanne) 14:1215356 37881498 10.3389/fendo.2023.1215356 PMC10597691

[24] Wilkie G, Leftwich HK (2021) Optimizing care preconception for women with diabetes and obesity. Clin Obstet Gynecol 64(1):226–233 33337741 10.1097/GRF.0000000000000590

[25] Chandrasekaran S, Neal-Perry G (2017) Long-term consequences of obesity on female fertility and the health of the offspring. Curr Opin Obstet Gynecol 29(3):180–187 28448277 10.1097/GCO.0000000000000364 PMC5983896

[26] Marshall NE, Abrams B, Barbour LA et al (2022) The importance of nutrition in pregnancy and lactation: lifelong consequences. Am J Obstet Gynecol 226(5):607–632 34968458 10.1016/j.ajog.2021.12.035 PMC9182711

[27] Giouleka S, Tsakiridis I, Koutsouki G et al (2023) Obesity in pregnancy: a comprehensive review of influential guidelines. Obstet Gynecol Surv 78(1):50–68 36607201 10.1097/OGX.0000000000001091

[28] Rozen G, Stern K, Lensen S, Polyakov A (2023) Barriers to reproductive treatments in Australia. Aust J Gen Pract 52(3):109–112 36872087 10.31128/AJGP-08-22-6551

[29] Boots CE, Gloff M, Lustik SJ, Vitek W (2024) Addressing weight bias in reproductive medicine: a call to revisit body mass index restrictions for in vitro fertilization treatment. Fertil Steril 122(2):204–210 38750875 10.1016/j.fertnstert.2024.05.140

[30] Rafael F, Rodrigues MD, Bellver J et al (2023) The combined effect of BMI and age on ART outcomes. Hum Reprod 38(5):886–894 36928306 10.1093/humrep/dead042

[31] Gautam D, Purandare N, Maxwell CV et al (2023) The challenges of obesity for fertility: a FIGO literature review. Int J Gynaecol Obstet 160(Suppl 1):50–55 36635080 10.1002/ijgo.14538 PMC10107441

[32] Goldberg AS, Dolatabadi S, Dutton H, Benham JL (2023) Navigating the role of anti-obesity agents prior to pregnancy: a narrative review. Semin Reprod Med 41(3–04):108–118 37973000 10.1055/s-0043-1776795

[33] TGA (2024) Prescribing medicines in pregnancy database

[34] Yang XD, Yang YY (2024) Clinical pharmacokinetics of semaglutide: a systematic review. Drug Des Devel Ther 18:2555–2570 38952487 10.2147/DDDT.S470826 PMC11215664

[35] Camilleri M (2015) Peripheral mechanisms in appetite regulation. Gastroenterology 148(6):1219–1233 25241326 10.1053/j.gastro.2014.09.016 PMC4369188

[36] Camilleri M, Lupianez-Merly C (2024) Effects of GLP-1 and other gut hormone receptors on the gastrointestinal tract and implications in clinical practice. Am J Gastroenterol 119(6):1028–1037 37753925 10.14309/ajg.0000000000002519 PMC11026296

[37] Drucker DJ (2018) Mechanisms of action and therapeutic application of glucagon-like peptide-1. Cell Metab 27(4):740–756 29617641 10.1016/j.cmet.2018.03.001

[38] Sathananthan A, Man CD, Micheletto F et al (2010) Common genetic variation in GLP1R and insulin secretion in response to exogenous GLP-1 in nondiabetic subjects: a pilot study. Diabetes Care 33(9):2074–2076 20805279 10.2337/dc10-0200 PMC2928367

[39] Camilleri M (2019) Gastrointestinal hormones and regulation of gastric emptying. Curr Opin Endocrinol Diabetes Obes 26(1):3–10 30418188 10.1097/MED.0000000000000448 PMC6615897

[40] Drucker DJ, Holst JJ (2023) The expanding incretin universe: from basic biology to clinical translation. Diabetologia 66(10):1765–1779 36976349 10.1007/s00125-023-05906-7

[41] Liu QK (2024) Mechanisms of action and therapeutic applications of GLP-1 and dual GIP/GLP-1 receptor agonists. Front Endocrinol (Lausanne) 15:1431292 39114288 10.3389/fendo.2024.1431292 PMC11304055

[42] Sumithran P, Prendergast LA, Delbridge E et al (2011) Long-term persistence of hormonal adaptations to weight loss. N Engl J Med 365(17):1597–1604 22029981 10.1056/NEJMoa1105816

[43] Mangliar I, Plante AS, Chabot M et al (2023) GLP-1 response during pregnancy: variations between trimesters and associations with appetite sensations and usual energy intake. Appl Physiol Nutr Metab 49(4):428–436 38095168 10.1139/apnm-2023-0301

[44] Qiao L, Lu C, Zang T, Dzyuba B, Shao J (2024) Maternal GLP-1 receptor activation inhibits fetal growth. Am J Physiol Endocrinol Metab 326(3):E268–E276 38197791 10.1152/ajpendo.00361.2023 PMC11193516

[45] Fandiño J, Toba L, González-Matías LC, Diz-Chaves Y, Mallo F (2019) Perinatal undernutrition, metabolic hormones, and lung development. Nutrients. 11(12):2870 31771174 10.3390/nu11122870 PMC6950278

[46] Nauck MA, Meier JJ (2018) Incretin hormones: their role in health and disease. Diabetes Obes Metab 20(Suppl 1):5–21 29364588 10.1111/dom.13129

[47] Drucker DJ (2022) GLP-1 physiology informs the pharmacotherapy of obesity. Mol Metab 57:101351 34626851 10.1016/j.molmet.2021.101351 PMC8859548

[48] Drucker DJ (2024) The benefits of GLP-1 drugs beyond obesity. Science 385(6706):258–260 39024455 10.1126/science.adn4128

[49] Pi-Sunyer X, Astrup A, Fujioka K et al (2015) A randomized, controlled trial of 3.0 mg of liraglutide in weight management. N Engl J Med 373(1):11–22 26132939 10.1056/NEJMoa1411892

[50] Garvey WT, Birkenfeld AL, Dicker D et al (2020) Efficacy and safety of liraglutide 3.0 mg in individuals with overweight or obesity and type 2 diabetes treated with basal insulin: the SCALE insulin randomized controlled trial. Diabetes Care 43(5):1085–1093 32139381 10.2337/dc19-1745 PMC7171937

[51] Wilding JPH, Batterham RL, Calanna S et al (2021) Once-weekly semaglutide in adults with overweight or obesity. N Engl J Med 384(11):989–1002 33567185 10.1056/NEJMoa2032183

[52] Karagiannis T, Malandris K, Avgerinos I et al (2024) Subcutaneously administered tirzepatide vs semaglutide for adults with type 2 diabetes: a systematic review and network meta-analysis of randomised controlled trials. Diabetologia 67(7):1206–1222 38613667 10.1007/s00125-024-06144-1 PMC11153294

[53] Jastreboff AM, Aronne LJ, Ahmad NN et al (2022) Tirzepatide once weekly for the treatment of obesity. N Engl J Med 387(3):205–216 35658024 10.1056/NEJMoa2206038

[54] Cesta CE, Rotem R, Bateman BT et al (2024) Safety of GLP-1 receptor agonists and other second-line antidiabetics in early pregnancy. JAMA Intern Med 184(2):144–152 38079178 10.1001/jamainternmed.2023.6663 PMC10714281

[55] Cena H, Chiovato L, Nappi RE (2020) Obesity, polycystic ovary syndrome, and infertility: a new avenue for GLP-1 receptor agonists. J Clin Endocrinol Metab 105(8):e2695-2709 32442310 10.1210/clinem/dgaa285 PMC7457958

[56] Catalano PM, Mele L, Landon MB et al (2014) Inadequate weight gain in overweight and obese pregnant women: what is the effect on fetal growth? Am J Obstet Gynecol 211(2):137.e131-137 10.1016/j.ajog.2014.02.004 PMC4117705 24530820

[57] Dao K, Shechtman S, Weber-Schoendorfer C et al (2024) Use of GLP1 receptor agonists in early pregnancy and reproductive safety: a multicentre, observational, prospective cohort study based on the databases of six Teratology Information Services. BMJ Open 14(4):e083550 38663923 10.1136/bmjopen-2023-083550 PMC11043712

[58] Jacqueline M, Pant D, Kaitlyn J et al (2024) Prepregnancy use of glucagon-like peptide 1 receptor agonist and gestational weight gain in Type 2 Diabetes. Diabetes 73:1214

[59] Price S, Sumithran P, Nankervis A, Permezel M, Prendergast L, Proietto J (2021) Impact of pre-conception weight loss on fasting glucose and pregnancy outcomes in women with obesity: a randomized trial. Obesity 29:1445–1457. 10.1002/oby.23200 34431233 10.1002/oby.23200

[60] Purcell K, Sumithran P, Prendergast LA, Bouniu CJ, Delbridge E, Proietto J (2014) The effect of rate of weight loss on long-term weight management: a randomised controlled trial. Lancet Diabetes Endocrinol 2(12):954–962 25459211 10.1016/S2213-8587(14)70200-1

[61] Pacifici GM, Nottoli R (1995) Placental transfer of drugs administered to the mother. Clin Pharmacokinet 28(3):235–269 7758253 10.2165/00003088-199528030-00005

[62] Fusco J, Xiao X, Prasadan K et al (2017) GLP-1/Exendin-4 induces β-cell proliferation via the epidermal growth factor receptor. Sci Rep 7(1):9100 28831150 10.1038/s41598-017-09898-4 PMC5567347

[63] Garcia-Flores V, Romero R, Miller D et al (2018) Inflammation-induced adverse pregnancy and neonatal outcomes can be improved by the immunomodulatory peptide exendin-4. Front Immunol 9:1291 29967606 10.3389/fimmu.2018.01291 PMC6015905

[64] Clarke GS, Li H, Ladyman SR, Young RL, Gatford KL, Page AJ (2024) Effect of pregnancy on the expression of nutrient-sensors and satiety hormones in mice. Peptides 172:171114 37926186 10.1016/j.peptides.2023.171114

[65] Negrato CA, Marques PR, Leite HB et al (2022) Glycemic and nonglycemic mechanisms of congenital malformations in hyperglycemic pregnancies: a narrative review. Arch Endocrinol Metab 66(6):908–918 36191262 10.20945/2359-3997000000521 PMC10118772

[66] Suarez-Roman G, Fernandez-Romero T, Perera-Calderin AJ, Rodriguez-Sosa VM, Arranz C, Hernandez SC (2016) Pregestational obesity-induced embryopathy. Reprod Sci 23(9):1250–1257 27089913 10.1177/1933719116635279

[67] Callaway LK, O’Callaghan MJ, McIntyre HD (2009) Barriers to addressing overweight and obesity before conception. Med J Aust 191(8):425–428 19835534 10.5694/j.1326-5377.2009.tb02876.x

[68] Endres LK, Sharp LK, Haney E, Dooley SL (2004) Health literacy and pregnancy preparedness in pregestational diabetes. Diabetes Care 27(2):331–334 14747209 10.2337/diacare.27.2.331

[69] Mittman BG, Le P, Payne JY, Ayers G, Rothberg MB (2024) Sociodemographic disparities in GLP-1RA and SGLT2i use among US adults with type 2 diabetes: NHANES 2005-March 2020. Curr Med Res Opin 40(3):377–383 38193509 10.1080/03007995.2024.2303413 PMC10947468

[70] Dimas A, Politi A, Papaioannou G et al (2022) The gestational effects of maternal appetite axis molecules on fetal growth, metabolism and long-term metabolic health: a systematic review. Int J Mol Sci 23(2):695 35054881 10.3390/ijms23020695 PMC8776066

[71] Conte C, Hall KD, Klein S (2024) Is weight loss-induced muscle mass loss clinically relevant? JAMA 332(1):9–10 38829659 10.1001/jama.2024.6586

[72] Nelson LW, Lee MH, Garrett JW et al (2024) Intrapatient changes in CT-based body composition after initiation of semaglutide (glucagon-like peptide-1 agonist) therapy. AJR Am J Roentgenol. 10.2214/AJR.24.31805 39230989 10.2214/AJR.24.31805

[73] Johansson K, Cnattingius S, Näslund I et al (2015) Outcomes of pregnancy after bariatric surgery. N Engl J Med 372(9):814–824 25714159 10.1056/NEJMoa1405789

[74] Wise J (2013) Women who have had surgery for obesity have raised risk of preterm babies. BMJ 347:f6774 24222654 10.1136/bmj.f6774

[75] Graham DL, Madkour HS, Noble BL, Schatschneider C, Stanwood GD (2021) Long-term functional alterations following prenatal GLP-1R activation. Neurotoxicol Teratol 87:106984 33864929 10.1016/j.ntt.2021.106984 PMC8555578

[76] Mahase E (2024) GLP-1 agonists: US sees 700% increase over four years in number of patients without diabetes starting treatment. BMJ 386:1645 10.1136/bmj.q1645 39043396

[77] Bouret SG, Simerly RB (2006) Developmental programming of hypothalamic feeding circuits. Clin Genet 70(4):295–301 16965320 10.1111/j.1399-0004.2006.00684.x

